# Determining the Clinical Value and Critical Pathway of GTPBP4 in Lung Adenocarcinoma Using a Bioinformatics Strategy: A Study Based on Datasets from The Cancer Genome Atlas

**DOI:** 10.1155/2020/5171242

**Published:** 2020-10-19

**Authors:** Zhiqian Zhang, Juan Wang, Jiayan Mao, Fangqiong Li, Wei Chen, Wei Wang

**Affiliations:** ^1^College of Medical Technology, Zhejiang Chinese Medical University, Hangzhou, Zhejiang 310053, China; ^2^Department of Clinical Laboratory, Tongde Hospital of Zhejiang Province, Hangzhou, Zhejiang 310012, China; ^3^Cancer Institute of Integrated Tradition Chinese and Western Medicine, Zhejiang Academy of Traditional Chinese Medicine, Tongde Hospital of Zhejiang Province, Hangzhou, Zhejiang 310012, China

## Abstract

Lung cancer is the leading cause of cancer-related death worldwide, and the most common histologic subtype is lung adenocarcinoma (LUAD). Due to the significant mortality and morbidity rates among patients with LUAD, the identification of novel biomarkers to guide diagnosis, prognosis, and therapy is urgent. Guanosine triphosphate-binding protein 4 (GTPBP4) has been found to be associated with tumorigenesis in recent years, but the underlying molecular mechanism remains to be elucidated. In the present study, we demonstrate that GTPBP4 is significantly overexpressed in LUAD primary tumors. A total of 55 genes were identified as potential targets of GTPBP4. GO enrichment analysis identified the top 25 pathways among these target genes, among which, ribosome biogenesis was shown to be the most central. Each target gene demonstrated strong and complex interactions with other genes. Of the potential target genes, 12 abnormally expressed candidates were associated with survival probability and correlated with GTPBP4 expression. These findings suggest that GTPBP4 is associated with LUAD progression. Finally, we highlight the importance of the role of GTPBP4 in LUAD *in vitro*. GTPBP4 knockdown in LUAD cells inhibited proliferation and metastasis, promoted apoptosis, and enhanced sensitivity to TP. Overall, we conclude that GTPBP4 may be considered as a potential biomarker of LUAD.

## 1. Introduction

Adenocarcinoma is the most prevalent subtype of lung cancer, comprising ~40% of all lung cancer cases [1, 2]. Despite a wealth of research, lung adenocarcinoma (LUAD) remains as a highly aggressive and fatal disease, with an overall survival time of <5 years due to the difficulty in diagnosis [2–4]. The discovery of novel specific molecular markers or technologies to diagnose LUAD is urgently required. Currently, computational biology is often combined with molecular biology and technology to explore the molecular mechanisms of disease and to identify clinically significant molecules [5]. Biomarkers, usually molecules involved in cancer development, play an important role in the diagnosis, treatment, and prognosis of various cancer types [6, 7].

Guanosine triphosphate-binding protein 4 (GTPBP4), also known as CRFG [8], NGB [9], and NOG1 [10], is a GTPase involved in the synthesis of 60S ribosomal subunit and located on nuclear chromosome 10p15-14 [11]. Previous studies had shown that GTPBP4 can induce cell proliferation and enhance cell colony formation in some cancer types [12, 13]. A study of colorectal carcinoma (CRC) also uncovered that GTPBP4 was responsible for tumor metastasis [14]. Meanwhile, patients with HCC (hepatocellular carcinoma) with high levels of GTPBP4 expression tended to have a poor prognosis [15]. The aforementioned research suggests that high expression of GTPBP4 is likely an important factor in the occurrence and development of tumors. However, there have been no prior reports regarding the expression or the role of GTPBP4 in lung adenocarcinoma.

In this study, we combined computational biology and experimental techniques to investigate the role of GTPBP4 in LUAD. The molecular function of GTPBP4 and its target genes were analyzed based on data from The Cancer Genome Atlas (TCGA) database. We demonstrate that the high expression of GTPBP4 contributes to LUAD tumorigenesis and might affect the prognosis of patients. Additionally, knockdown of GTPBP4 in A549 and H1299 cells inhibits proliferation and migration and improves cell apoptosis. Therefore, this study may provide a novel molecular target for the treatment of LUAD.

## 2. Materials and Methods

### 2.1. GTPBP4 Expression in LUAD

The Cancer Genome Atlas (TCGA) is a high-throughput gene database containing data regarding >30 types of human carcinoma [16]. We obtained the GTPBP4 expression profiles for diverse cancer types and with respect to gender, node metastasis status, ethnicity, and stage, based on a TCGA online analysis tool (http://ualcan.path.uab.edu/index.html).

### 2.2. Prediction and Data Screening of GTPBP4 Target Genes

A total of 5 programs, including String (https://string-db.org/), BioGRID (https://thebiogrid.org/), BioPlex (https://bioplex.hms.harvard.edu/), HPRD (http://www.hprd.org), and InBio_Map (https://www.intomics.com/inbio/map/#home), were used to identify the target genes of GTPBP4. The target genes that overlapped in ≥3 of 5 programs were selected to improve the accuracy of the results. The integration of the genes was visualized by a Venn diagram (https://www.omicshare.com/tools/).

### 2.3. Functional Annotation of the Selected Target Genes in LUAD

Gene Ontology (GO) enrichment analysis was performed to uncover the biological function of the overlapping target genes of GTPBP4 in LUAD. The online analysis tool (https://www.omicshare.com/tools/) was applied to explore the significance of the overlapping target genes of GTPBP4.

### 2.4. Protein-Protein Interaction (PPI) Network Construction

The retrieval of interacting genes search tool (http://metascape.org/gp/index.html#/main/step1) was used to diagram the PPI network of the overlapping target genes.

### 2.5. Identification of Differentially Expressed Genes

The expression of the overlapping genes between LUAD primary tumor and adjacent normal tissues was analyzed using a TCGA data online analysis tool (http://ualcan.path.uab.edu/index.html). Results with statistical differences (*P* < 0.05) were recorded as differentially expressed genes.

### 2.6. Assessment of the Prognostic Value of the Overlapping Genes of GTPBP4

The overlapping genes were searched on TCGA data online analysis tool (http://ualcan.path.uab.edu/index.html) to retrieve the associated survival curves (*P* < 0.05).

### 2.7. Correlation Analysis of GTPBP4 and the Selected Target Genes

We used another TCGA analysis tool (http://gepia.cancer-pku.cn/detail.php) to define the correlation between GTPBP4 and the selected overlapping genes.

### 2.8. Cell Lines and Cultures

Human LUAD cell lines (A549 and NCI-H1299) were purchased from the American Type Culture Collection (ATCC; VA, USA) and cultured in RPMI 1640 (Roswell Park Memorial Institute 1640) medium (Gibco, Grand Island, NY) containing 1% penicillin/streptomycin (Sigma, St Louis, MO) and 10% fetal bovine serum (FBS, Gibco). All cells were maintained at 37°C in 5% CO_2_.

### 2.9. Cell Transfection

GTPBP4 small interfering RNA (siRNA) and negative control siRNA were synthesized obtained from GenePharma Co. (Shanghai, China). Briefly, cells at a density of 2.0 × 10^5^ cells per well were transfected with siRNA using Lipofectamine 2000 (Invitrogen, Carlsbad, CA) in a serum-free medium in a 6-well plate. After 6 hours, the medium was replaced with fresh medium containing FBS. All subsequent experiments were performed at least 24 h after transfection. The sequences of the GTPBP4 siRNA are as follows: GTPBP4-homo-245: sense: 5′-CCAUGAUAGACUUUCACAATT-3′ and antisense: 5′-UUGUGAAAGUCUAUCAUGGTT-3′; GTPBP4-homo-868: sense: 5′-GGGAGCAGCUAGAACUCUUTT-3′ and antisense: 5′-AAGAGUUCUAGCUGCUCCCTT-3′; GTPBP4-homo-1374: sense: 5′-GGCCAUAAUAUAGCUGAUUTT-3′ and antisense: 5′-AAUCAGCUAUAUUAUGGCCTT-3′.

### 2.10. Cell Viability Assay

LUAD cells were seeded into 96-well plates at a density of 5 × 10^3^ cells per well and incubated overnight. Then, the medium was replaced with media containing different concentrations of Triptolide (TP; Sigma-Aldrich) (0, 0.78125, 1.5625, 3.125, 6.25, 12.5, and 25 ng/mL) and incubated with the cells for 48 h. Cell viability was estimated using a Cell Counting Kit-8 assay (CCK8; Dojindo; Kumamoto, Japan), according to the manufacturer's instruction. A MAX II microplate reader (Dynex Technologies, Chantilly, VA) was used to measure the absorbance at 450 nm. The half maximal inhibitory concentration (IC_50_) of TP was measured using the following equation: *V*% = 100/(1 + 10^[TP]logIC50^), where *V*% is the percentage viability and [TP] is the TP concentration (ng/mL).

### 2.11. 5-Ethynyl-2′-deoxyuridine (EdU) Incorporation Assay

DNA synthesis was quantified using a Click-iT™ EdU Imaging kit (Invitrogen; Carlsbad, CA, USA) according to the manufacturer's instruction. Briefly, cells were seeded in 96-well plates and 10 *μ*M EdU was added to culture for 2 h. Next, cells were fixed with 4% paraformaldehyde for 15-30 min and permeabilized with 0.5% Triton X-100 for 20 min at room temperature. After washing with PBS, 100 *μ*L Click-iT reaction mixture was incubated with the cells for 30 min, followed by 100 *μ*L Hoechst 33342 in PBS for 30 min. The results were visualized using a NanoZoomer 2.0-RS fluorescence microscope (Hamamatsu, Japan).

### 2.12. Cell Apoptosis Analysis

Preconditioned A549 and H1299 cells were harvested and then washed twice with ice-cold PBS. Then, Annexin V-FITC and PI dyes (BD Biosciences, Franklin Lakes, NJ, USA) were used to stain the cells, according to the manufacturer's description. Cells were finally analyzed via flow cytometry (BD Biosciences, Franklin Lakes, NJ, USA).

### 2.13. Western Blotting

Cells were washed twice with PBS and transferred to 100 *μ*L lysis buffer (Beyotime Co, China) containing 100 mM phenylmethanesulfonyl fluoride (PMSF, Beyotime Co, China), in which they were incubated for 30 min on ice. The soluble protein fractions were collected after centrifugation at 12000 × g for 20 min at 4°C, and protein concentration was quantified using a BCA protein assay kit (Thermo Fisher; Rockford, IL, USA), according to the manufacturer's protocol. Equal amounts of protein were denatured and separated by 10% SDS-PAGE and then transferred into polyvinylidene difluoride (PVDF) membranes (Millipore; Billerica, MA, USA). PVDF membranes were blocked with 5% nonfat milk in TBST (Tris-buffered saline (TBS) containing 0.1% Tween 20) at room temperature for 2 hours. Membranes were then incubated with anti-GTPBP4 (13897-1-AP; Proteintech, Wuhan, China) and anti-GAPDH (2118S; CST, Danvers, MA, USA), diluted 1 : 1000 in TBST overnight at 4°C. The membranes were then washed 3 times and incubated with a horseradish peroxidase-conjugated secondary antibody, diluted 1 : 2000 in TBST, at room temperature for 2 hours. Signals were visualized using ECL reagents (Thermo Scientific, Waltham, MA, USA), using GAPDH as a loading control.

### 2.14. Scrape Motility Assay

The scrape motility assay was used to evaluate cell migration. Cells (3.5 × 10^5^ per well) were seeded in 6-well plates. Once the cells had formed confluent monolayers, a 200 *μ*L sterile pipette tip was used to create a scratch in each well. The floating cells were removed, and the anchorage-dependent cells were incubated in serum-free medium. Images were captured under an inverted light microscope (Olympus IX51, Olympus, Center Valley, PA, USA) at 0, 24, and 48 hours after scratching.

### 2.15. Statistical Analysis

Each experiment was performed independently ≥3 times. Results are presented as mean ± standard deviation (SD). All data were analyzed using GraphPad Prism (version 8; GraphPad, San Diego, CA). Student's *t* test was used to analyze differences between groups, and *P* < 0.05 was considered to indicate a statistically significant difference.

## 3. Results

### 3.1. GTPBP4 Expression in LUAD Tissues

**Figure 1:**
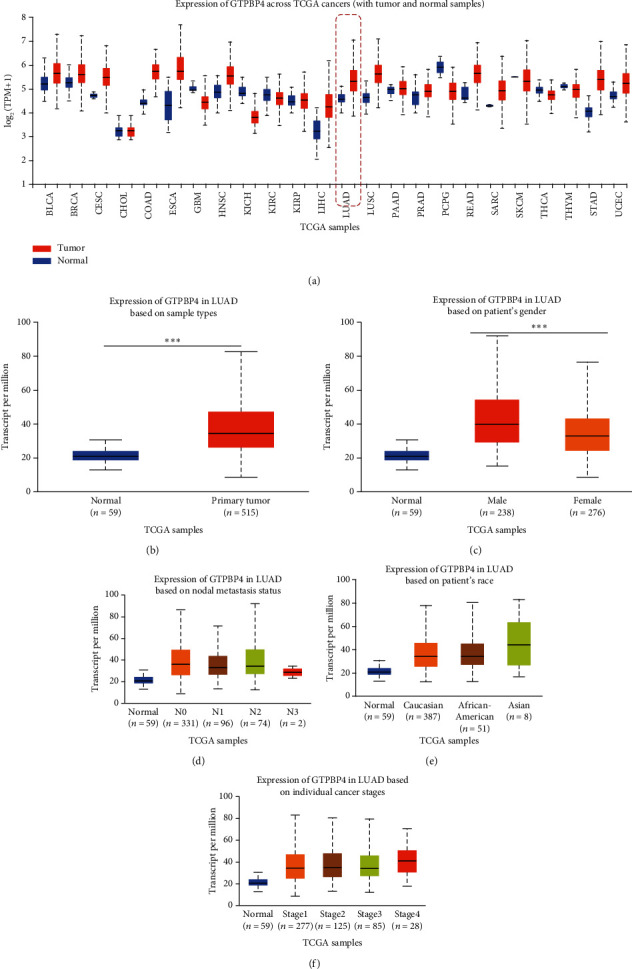
The expression profile of GTPBP4 in LUAD clinical tissues. (a) GTPBP4 is overexpressed in the majority of human cancer types available via TCGA, including LUAD. (b) GTPBP4 expression is increased in primary tumor LUAD samples compared to normal samples. ∗∗∗*P* < 0.001. (c) The expression of GTPBP4 in males and females with LUAD. (d) The expression of GTPBP4 according to node metastasis status in patients with LUAD. N0: no regional lymph node metastasis; N1: metastases in 1-3 axillary lymph nodes; N2: metastases in 4-9 axillary lymph nodes; N3: metastases in ≥10 axillary lymph nodes. (e) The expression of GTPBP4 according to ethnicity among patients with LUAD. (f) The expression of GTPBP4 according to LUAD stage.

To confirm the expression level of GTPBP4 in various human tumors, we used UALCAN, a TCGA data online analysis tool, to derive the GTPBP4 expression profiles. GTPBP4 expression was increased in most human cancer types when compared with adjacent normal tissues (Figure 1(a)), and this difference was significant in LUAD primary tumor tissues (Figure 1(b)). GTPBP4 expression profiles in LUAD tissues were also compared for gender (male and female), node metastasis status (N0, N1, N2, and N3), ethnicity (Caucasian, African-American, and Asian), and stage (S1, S2, S3, and S4). Statistical analysis revealed that, in patients with LUAD, GTPBP4 expression was higher in men than in women (Figure 1(c)). Excluding the impact of sample size, node metastasis status, ethnicity, and tumor stage did not result in the differential expression of GTPBP4 (Figures 1(d)–1(f)).

### 3.2. Prediction and Data Screening of GTPBP4 Target Genes

**Figure 2:**
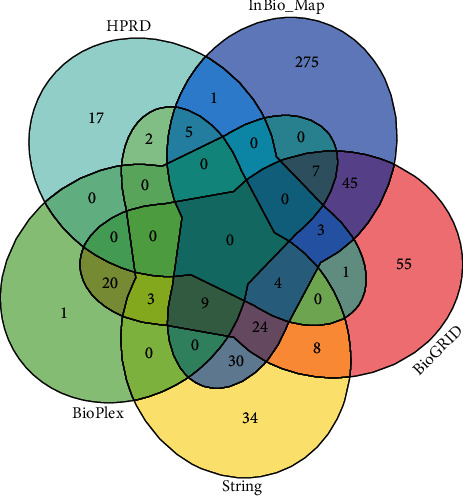
Venn diagram of the predicted target derived from 5 databases.

Groups of potential GTPBP4 target genes were selected from 5 public databases. A total of 119 genes were obtained from String, 179 from BioGRID, 40 genes from BioPlex, 403 from InBio_Map, and 33 from HPRD. This resulted in a total of 774 potential target genes. To improve prediction accuracy, only genes obtained from ≥3 of 5 databases (overlapping genes) were selected as potential targets of GTPBP4 for further analysis, which was a total of 55 genes (Figure 2).

### 3.3. Functional Analysis of the Overlapping GTPBP4 Target Genes in LUAD

**Figure 3:**
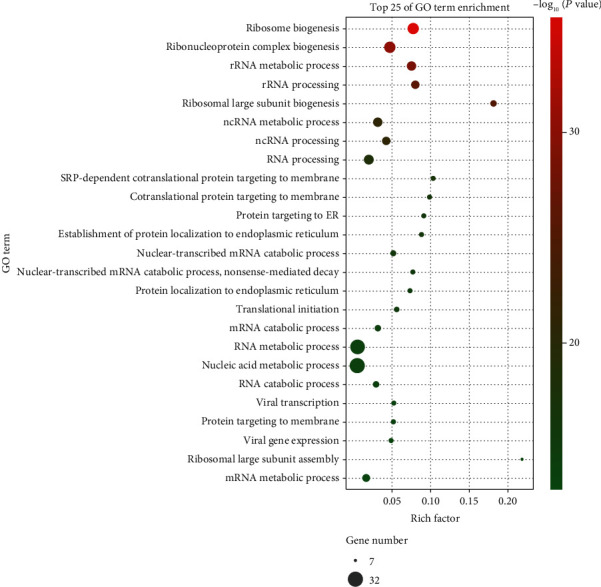
GO enrichment analysis of the identified overlapping target genes of GTPBP4 in LUAD.

The functional roles of 55 potential target genes in LUAD were analyzed in terms of biological processes (BP) by GO enrichment analysis. The top 25 enriched pathways with significant differences were selected to construct a bubble chart (Figure 3). The biological functions of GTPBP4 target genes were mainly associated with RNA processing and metabolism, and the most significant pathway was ribosome biogenesis (*P* < 0.001).

### 3.4. Protein-Protein Interaction (PPI) Enrichment Analysis of the Overlapping Target Genes

**Figure 4:**
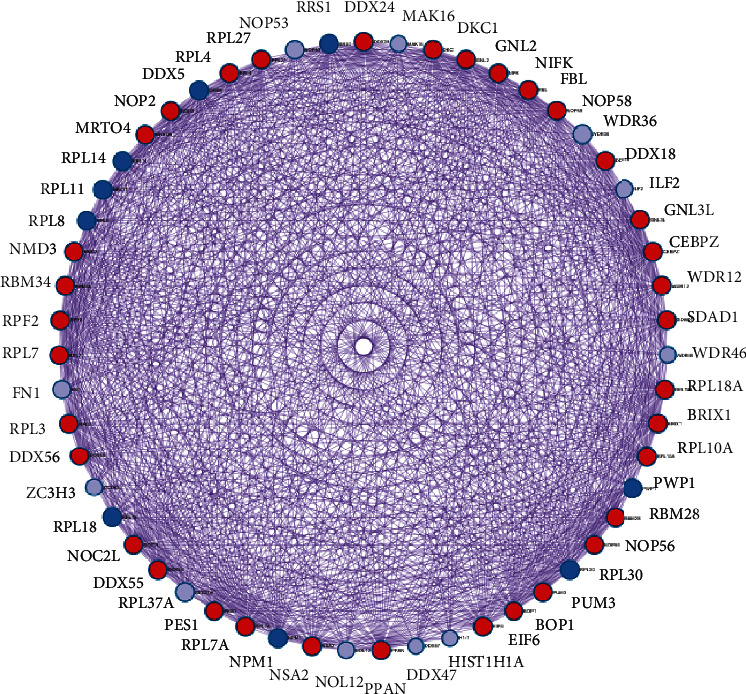
PPI network of the identified overlapping genes of GTPBP4.

To determine the interaction among the proteins encoded by the overlapped genes in LUAD, Metascape software was used to construct a PPI network. This was performed using 3 databases: BioGRID, InWeb_IM, and OmniPath. As indicated in Figure 4, the interactions among target genes are complicated, and each target gene has intricate associations with other genes. Most of the targets belong to the PRL family, the DDX family, and the NOP family; these families all were reported to be essential for ribosome biogenesis and RNA metabolism, and this corresponds with the results in Section 3.3.

### 3.5. Validation of the Overlapping Target Genes of GTPBP4 in LUAD from TCGA Data

**Figure 5:**
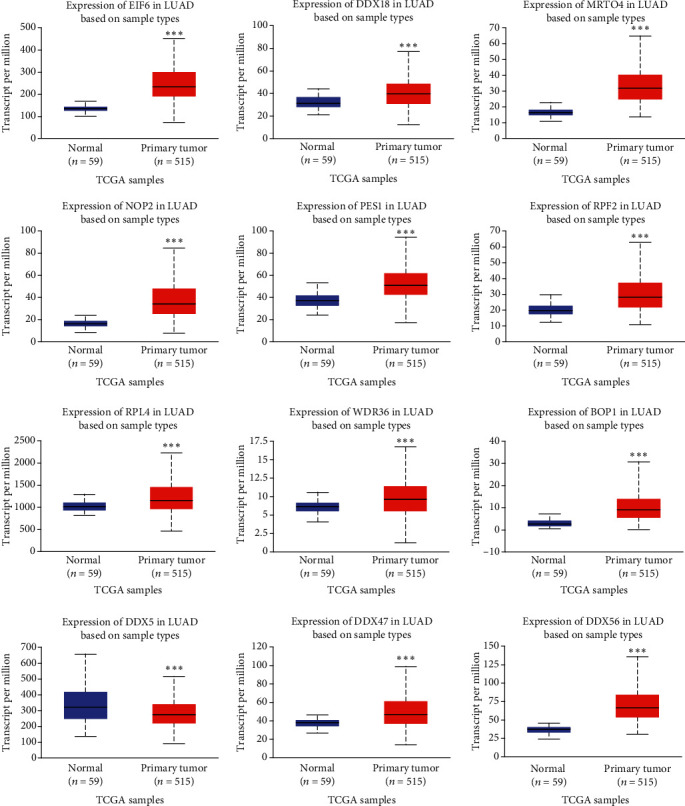
Expression of the 12 genes in LUAD identified using UALCAN. The blue node represents normal samples, and the red node represents tumor samples. The expression of EIF6, DDX18, MRTO4, NOP2, PES1, RPF2, RPL4, WDR36, BOP1, DDX47, and DDX56 is increased in LUAD compared to normal control, whereas the expression of DDX5 was decreased in LUAD. ∗∗∗*P* < 0.001.

**Figure 6:**
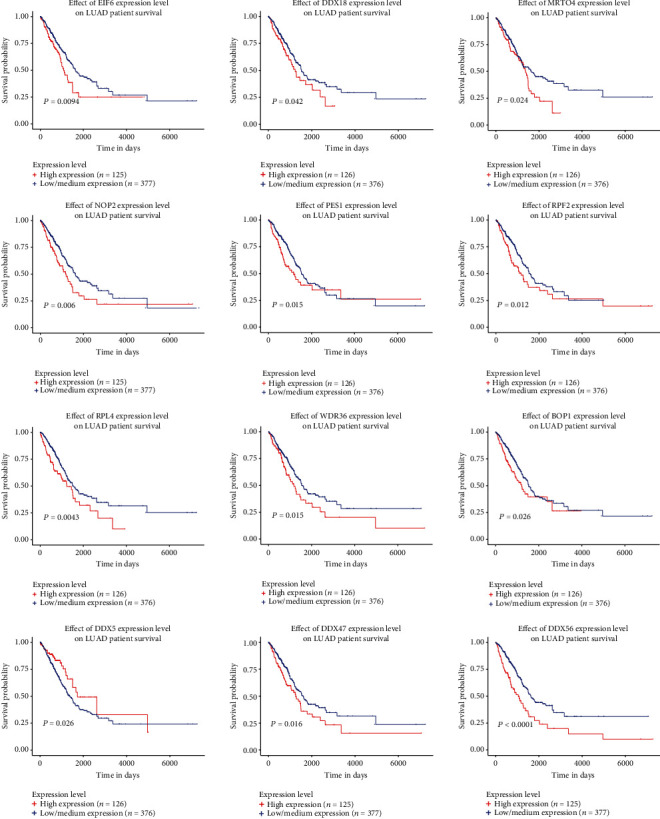
The prognostic significance of the 12 selected target genes for LUAD. Low expression of DDX5 and high expression of EIF6, DDX18, MRTO4, NOP2, PES1, RPF2, RPL4, WDR36, BOP1, DDX47, and DDX56 were associated with poor prognosis of LUAD patients.

**Figure 7:**
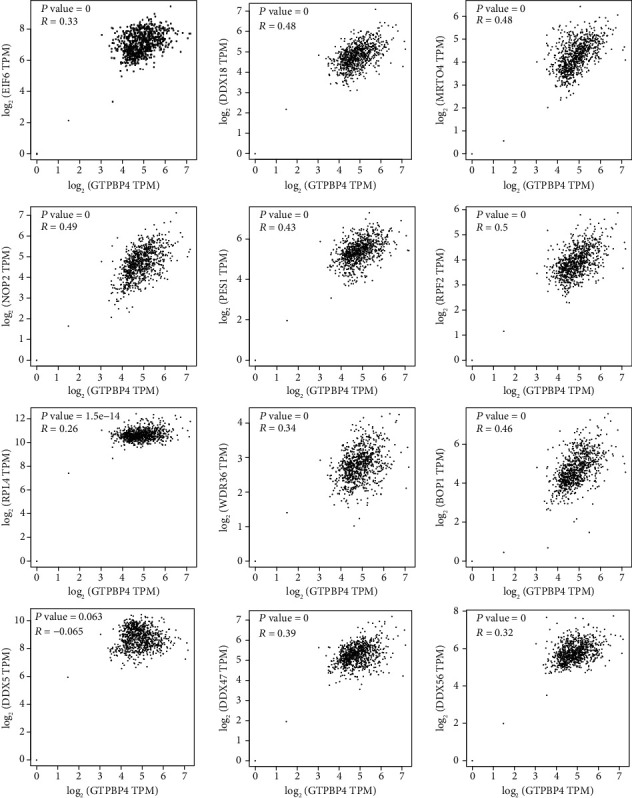
Validation of the 12 selected target genes using the GEPIA server. EIF6, DDX18, MRTO4, NOP2, PES1, RPF2, RPL4, WDR36, BOP1, DDX47, and DDX56 all positively correlated with GTPBP4 in LUAD, whereas DDX5 was negatively correlated with GTPBP4.

To further explore the role of GTPBP4 in LUAD, the expression of the overlapping target genes and their association with prognosis were analyzed using the UALCAN program. According to the query results, 12 genes with the most significant association between expression and prognosis in LUAD were selected for further analysis. Among the 12 genes (NOP2, DDX18, EIF6, BOP1, PES1, DDX47, RPF2, DDX56, MRTO4, RPL4, DDX5, and WDR46), all were upregulated in LUAD tissues compared to normal adjacent tissue except for DDX5 (Figure 5). In terms of LUAD prognosis, patients with downregulated DDX5 expression, or overexpression of NOP2, DDX18, EIF6, BOP1, PES1, DDX47, RPF2, DDX56, MRTO4, RPL4, and WDR46, had lower survival probabilities (Figure 6). Furthermore, the correlation between the expression of GTPBP4 and these 12 genes was calculated using GEPIA. In patients with LUAD, NOP2, DDX18, EIF6, BOP1, PES1, DDX47, RPF2, DDX56, MRTO4, RPL4, and WDR46 had a positive correlation with GTPBP4, while DDX5 had a negative correlation with GTPBP4 (Figure 7).

### 3.6. Knockdown of GTPBP4 Suppressed Cell Proliferation and Accelerated Cell Apoptosis of LUAD Cells

**Figure 8:**
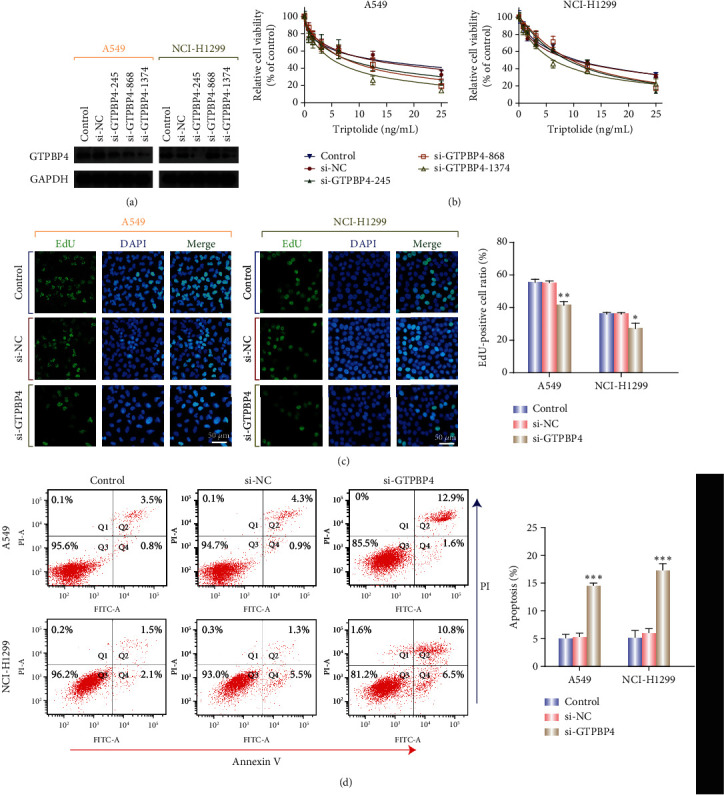
GTPBP4 regulates LUAD cell proliferation and apoptosis. (a) Knockdown of GTPBP4 in A549 and H1299 cells using si-NC, si-GTPBP4-245, si-GTPBP4-868, and si-GTPBP4-1374 was confirmed by western blotting (left: A549 cells; right: H1299 cells). (b) Viability of A549 and H1299 cells was determined under different concentrations of TP (25, 12.5, 6.25, 3.125, 1.5625, 0.78125, and 0 ng/mL). (c) Proliferation rates of LUAD cells were analyzed using the EdU assay with an IC_50_ concentration of TP. ∗*P* < 0.05 and ∗∗*P* < 0.01. (d) Cell apoptosis was evaluated by Annexin V staining; mean ± SD (*n* = 3), ∗∗∗*P* < 0.001.

To verify the results of bioinformatics analysis, we analyzed the effect of downregulated GTPBP4 expression in LUAD cells *in vitro*. Western blotting showed that siRNA targeting GTPBP4 (si-GTPBP4-245, si-GTPBP4-868, and si-GTPBP4-1374) markedly suppressed the expression of GTPBP4 in both A549 and H1299 cells. The most striking downregulation of GTPBP4 was achieved by si-GTPBP4-1374 (Figure 8(a)). CCK8 assays showed that RNA interference of GTPBP4 significantly increased the sensibility of A549 and H1299 cells to TP compared to control cells (Figure 8(b)). Based on the results of western blotting and CCK8 assays, si-GTPBP4-1374 was selected for use in subsequent experiments. The EdU assays revealed that proliferation of A549 and H1299 cells was suppressed by si-GTPBP4 (Figure 8(c)). Similarly, downregulation of GTPBP4 expression induced apoptosis of both A549 and H1299 cells (Figure 8(d)).

### 3.7. GTPBP4 Facilitates the Migration of LUAD Cells

**Figure 9:**
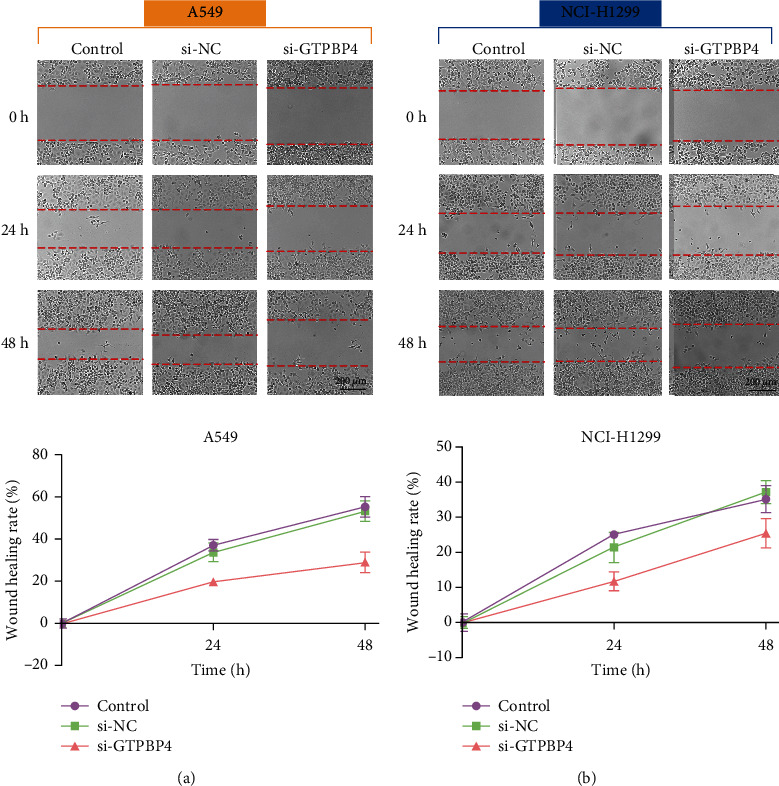
GTPBP4 promotes migration of LUAD cells. Wound healing was monitored for 48 h in A549 and H1299 cell monolayers; mean ± SD (*n* = 3).

To investigate whether GTPBP4 may contribute to LUAD metastasis, siRNA was used to downregulate GTPBP4 expression in A549 and H1299 cells. Downregulation of GTPBP4 expression in A549 cells partially inhibited the migration of LUAD cells 24 h posttransfection, and this inhibition was magnified at 48 h after transfection. In H1299 cells, downregulated GTPBP4 expression also repressed metastasis 24 h and 48 h posttransfection compared with the control and si-NC groups. There was no difference in metastasis between the control and si-NC groups. These results indicated that GTPBP4 facilitated the migration of LUAD cells (Figure 9).

## 4. Discussion

GTPBP4 is a molecular switch that is of great importance for the biogenesis of the 60S ribosomal subunit and signal transmission due to its GTPase activity [8, 17, 18]. It switched between an active state and inactive state when bound with GTP or GDP [19]. At a time when targeted therapy is popular in the field of cancer research, GTBBP4 has been identified as a potential biomarker for various cancer types. However, recent studies have indicated that the role of GTPBP4 in malignant tumor types is double sided. Although GTPBP4 has been most often found to act as an oncogene [12, 13], it has also been described as a suppressor gene in rare cases, such as in neurofibromatosis 2 (NF2) [9], suggesting that the role of GTPBP4 depends on the specific type of cancer.

In the present study, we have demonstrated that the expression of GTPBP4 is higher in tumor tissues than in adjacent normal control. Similarly, the immunohistochemistry (IHC) database of HPA (https://www.proteinatlas.org) showed that the protein expression of GTPBP4 in LUAD tissues was increased compared to normal control (Figure S1). In addition, to further investigate the function of GTPBP4, its potential downstream target genes were identified. We show that GTPBP4 may be a novel potential biomarker to guide LUAD diagnosis, prognosis, and treatment. Suppression of GTPBP4 expression in A549 and H1299 cells inhibited cell proliferation and migration and increased the rate of apoptosis. Based on the anti-tumor activity of TP and its toxicity to LUAD cells [20], our study indicated that RNA interference of GTPBP4 in A549 and H1299 cells significantly increased sensibility to TP.

Some studies have reported that GTPBP4 plays a critical role in the progression of tumors. For example, aberrantly expressed GTPBP4 was found to be significantly associated with low survival probability in patients with HCC and breast cancer [15, 21]. Yu et al. concluded that GTPBP4 was responsible for tumor metastasis in CRC [14], and Li et al. suggested that GTPBP4 promotes gastric cancer progression [12]. The analysis of its potential target genes in the present study suggested that GTPBP4 may be a predictor of survival in LUAD cases. The overall survival analysis graph indicates that high expression of GTPBP4 in LUAD is associated with poor prognosis; however, this was not statistically significant (Figure S2). This also implies that GTPBP4 may predict prognosis of patients with LUAD.

As shown in Figure 3, ribosome biogenesis was predicted to be the crucial biological process involving GTPBP4. Importantly, a series of studies have indicated that dysregulated ribosome biogenesis is essential for the tumorigenesis of most spontaneous cancer types and that ribosome biogenesis is closely related to tumor suppressor P53 in cell proliferation and apoptosis [22–24]. The majority of the selected target genes in the present study were also observed to participate in ribosome synthesis. EIF6 (eukaryotic initiation factor 6) has been shown to be essential for nucleolar biogenesis of 60S ribosomes and maximal protein synthesis downstream of growth factor stimulation [25]. The assembly of 40S and 60S ribosomal subunits is regulated by DEAD-box RNA helicase 18 (DDX18) [26]. Nucleolar protein 2 (NOP2/NSUN1), which can inhibit HIV-1 transcription and promote viral latency [27], is also required for nucleolar maturation and ribosome biogenesis in mammals [28]. Ribosomal protein L4 (RPL4) is known to affect tumorigenesis and metastasis of HCC [29] and to participate in the assembly of pre-60S in the nucleus [30]. BOP1 (Block of Proliferation 1) is responsible for modulating pre-rRNA processing of 28S and 5.8S rRNAs [31, 32]. Additionally, DEAD-box RNA helicase 56 (DDX56) is involved in the assembly of the 60S large ribosomal subunit and has been associated with lymphatic invasion and distant metastasis in CRC [33]. Pescadillo ribosomal biogenesis factor 1 (PES1), which has been reported as an independent poor prognostic factor in pancreatic cancer patients [34], is essential for 60S ribosomal subunit maturation and pre-rRNA processing [35]. Therefore, we speculated that the regulation of ribosome biogenesis by GTPBP4 plays an essential role in various types of cancer. Regarding promoted progression of gastric cancer by GTPBP4 regulation of P53 activity [12], we infer that aberrant GTPBP4 affects LUAD cell proliferation, apoptosis, and migration by disturbing the balance between ribosome biogenesis and P53 activity. Thus, GTPBP4 is likely to participate in LUAD by regulating ribosomal biogenesis.

## 5. Conclusions

In conclusion, our findings suggest that aberrantly high expression of GTPBP4 contributes to the tumorigenesis of LUAD and that it may be associated with prognosis of patients with LUAD. GTPBP4 may have potential as a novel therapeutic target, a diagnostic biomarker, or a survival predictor for LUAD.

## Data Availability

The data used to support the findings of this study are available from the corresponding author upon request.

## Supplementary Materials

Supplementary Materials Figure S1: IHC of GTPBP4 in LUAD sample and normal lung sample from HPA, ×100 magnification. Figure S2: survival curve analysis based on GTPBP4 expression in patients with LUAD; the high expression of GTPBP4 was associated with poor prognosis of LUAD patients (*P* = 0.07).
