# Supplementary material for: Determining the Clinical Value and Critical Pathway of GTPBP4 in Lung Adenocarcinoma Using a Bioinformatics Strategy: A Study Based on Datasets from The Cancer Genome Atlas
Source: Biomed Res Int. 2020 Oct 19;2020:5171242. doi: 10.1155/2020/5171242 (PMC7593728; doi:10.1155/2020/5171242)
Supplement: Supplementary Materials — Figure S1: IHC of GTPBP4 in LUAD sample and normal lung sample from HPA, ×100 magnification. Figure S2: survival curve analysis based on GTPBP4 expression in patients with LUAD; the high expression of GTPBP4 was associated with poor prognosis of LUAD patients (P = 0.07). [file 5171242.f1.pdf]

6. Supplementary Materials

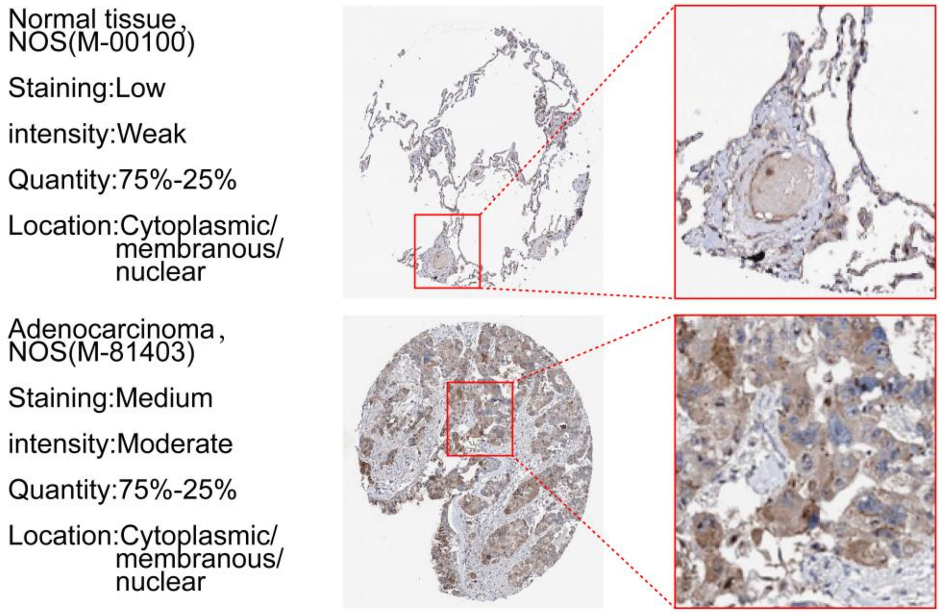


Figure S 1 IHC of GTPBP4 in LUAD sample and normal lung sample from HPA. × 200 magnification.

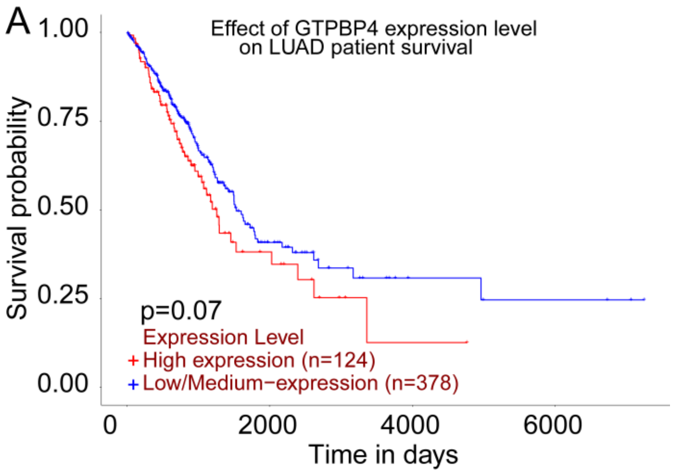


Figure S 2 Survival curve analysis based on GTPBP4 expression in patients with LUAD.

**Data Availability**

The data used to support the findings of this study are available from the corresponding author upon request.

**Conflicts of Interest**

The authors declare that there is no conflict of interest regarding the publication of this paper.
